# Tunable polytetrafluoroethylene electret films with extraordinary charge stability synthesized by initiated chemical vapor deposition for organic electronics applications

**DOI:** 10.1038/s41598-018-38390-w

**Published:** 2019-02-19

**Authors:** Stefan Schröder, Thomas Strunskus, Stefan Rehders, Karen K. Gleason, Franz Faupel

**Affiliations:** 1 Institute for Materials Science, Christian-Albrechts-Universität zu Kiel, 24143 Kiel, Germany; 2 Department of Chemical Engineering, Massachusetts Institute of Technology, Cambridge, MA 02139 USA

## Abstract

Bulk polytetrafluoroethylene (PTFE) possesses excellent chemical stability and dielectric properties. Indeed, thin films with these same characteristics would be ideal for electret applications. Previously, the electret properties of PTFE-like thin films produced by rf sputtering or plasma enhanced chemical vapor deposition were found to deteriorate due to structural changes and surface oxidation. In this article, the technique of initiated chemical vapor deposition (iCVD) is evaluated for electret applications for the first time. The iCVD method is known for its solvent-free deposition of conformal, pinhole-free polymer thin films in mild process conditions. It is shown that PTFE thin films prepared in this way, show excellent agreement to commercial bulk PTFE with regard to chemical properties and dielectric dissipation factors. After ion irradiation in a corona discharge the iCVD PTFE thin films exhibit stable electret properties, which can be tailored by the process parameters. Due to the mild deposition conditions, the iCVD technique is suitable for deposition on flexible organic substrates for the next-generation electret devices. It is also compatible with state-of-the-art microelectronic processing lines due to the characteristics of conformal growth and easy scaling up to larger size substrates.

## Introduction

**Figure 1:**
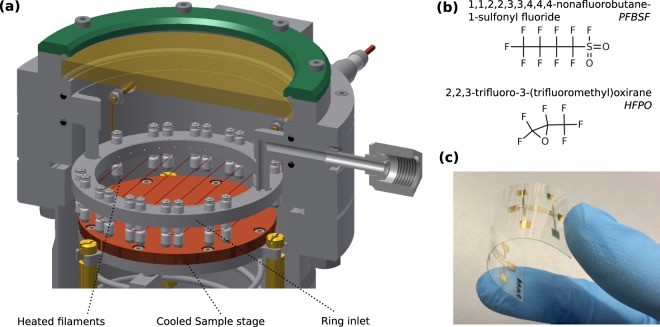
**(a)** Illustration of the radial-flow type reactor used in this study. The gas is supplied through the ring inlet to the reactor and the sample stage is cooled to room temperature. The system is operated in continuous flow mode. **(b)** Structural formulas of the initiator molecule perfluorobutanesulfonyl flouride (PFBSF, 1,1,2,2,3,3,4,4,4-nonafluorobutane-1-sulfonyl fluoride) and the monomer molecule hexafluoropropylene oxide (HFPO, 2,2,3-Trifluoro-3-(trifluoromethyl)oxirane)) used for the deposition of PTFE thin films. **(c)** Photograph of a flexible substrate with DC magnetron sputtered gold electrode structures and iCVD polymer thin film dielectric.

Besides its excellent chemical stability and dielectric properties polytetrafluoroethylene (PTFE) is well known for its outstanding performance as a real charge electret material^1–3^. An electret is a dielectric material with a quasipermanent dipole polarization or surface charge, the latter one usually generated by ion irradiation in a corona discharge or electron beam irradiation^3^. It can deliver a built-in potential in portable electronic devices to power it without external bias voltage. The best known application is probably the millionfold produced electret condenser microphone for mobile electronic devices, like mobile phones and hearing aids^4,5^. Various other applications take advantage of electrets ranging from electrostatic air filters to organic transistor memory and electret memristors^6–8^. Most recently the increasing interest in electret generators^9–13^ demands furthermore high performance electrets, often on flexible substrates^11–13^. For next-generation electret devices, a fluoropolymer thin film deposition technique is highly demanded to enable device miniaturization, precise fluoropolymer thin film control, as well as the integration into state-of-the-art industrial microelectronic processing lines. Particularly for PTFE electrets, high quality PTFE thin films are needed, that preserve the original nature of PTFE. Some promising approaches have been reported^14–16^. Nevertheless corona-charged PTFE thin films deposited, e.g. by radio frequency (rf) sputtering show significant differences in charge storage and electrical properties compared to commercial PTFE^14^, due to deviations from the original PTFE structure. Also much higher dissipation factors are reported for rf sputtered PTFE thin films^17^. PTFE thin films prepared by plasma polymerization suffer often from C=O group formation due to oxidation which increases also the dissipation factor of the film^18^ and consequently impairs the electret properties^15^. Promising PTFE film fabrication approaches with pulsed laser deposition (PLD)^16^ are not suitable for large-area deposition. High temperatures are furthermore involved in most of these approaches and are thus not suitable for sensitive flexible polymer substrates used, e.g. in next generation flexible organic electronic devices like wearable electret generators^11–13^. In this approach, the mentioned problems are solved by using PTFE thin films synthesized via initiated chemical vapor deposition (iCVD). This technique is known to preserve the original polymer functionality and to maintain a low substrate temperature, due to its mild deposition conditions^19,20^, allowing thus the deposition on flexible organic susbtrates. The iCVD process is already well established for PTFE thin film deposition^21–23^. The technique does not involve any organic solvents, because the thin film is directly polymerized by a free radical polymerization on a substrate, which is cooled to room temperature. An initiator provides CF_3_ end groups, preventing polar group formation at dangling bonds, and increases thus the dielectric performance regarding, e.g. dissipation factors. It furthermore increases the deposition rate significantly. Rates of several µm h^−1^ are reported for iCVD PTFE^21,23^, so that the demanded film thickness of typically 10 µm for stable polymer electrets is easily obtained in this way. The CVD-typical conformal growth enables furthermore smooth film surfaces and easy scale-up of the process to larger dimension substrates, making it interesting for industrial applications^19,23^. The PTFE thin film electrets in this study were fabricated in a custom-made, hot filament, radial-flow-type reactor with ring-inlet, as illustrated in Fig. 1a.

**Figure 2:**
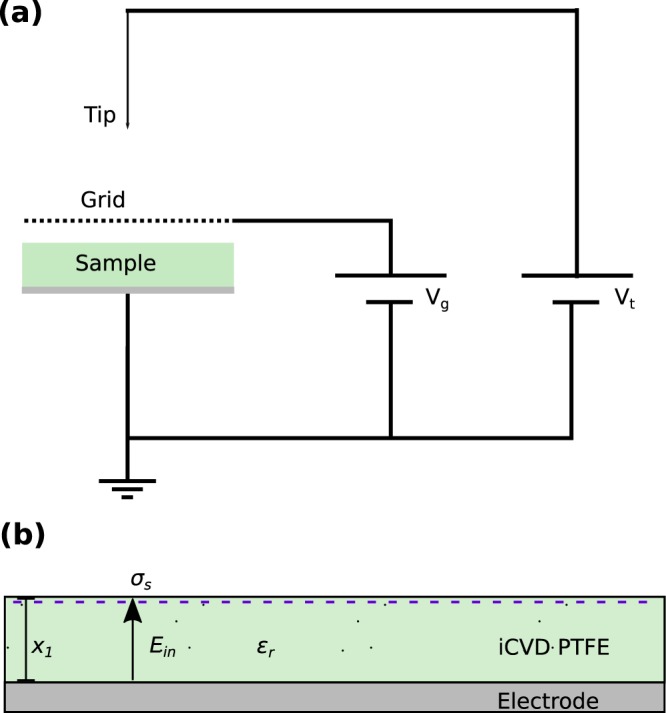
**(a)** Schematic of a corona discharge setup. The tip potential *V*_*t*_ and optional grid with grid potential *V*_*g*_ are applied to generate the surface charge by ion irradiation. **(b)** Cross-sectional view of the PTFE thin film after the ion irradiation. Due to the surface charge (*σ*_*s*_), the electret establishes an internal electric field *E*_*in*_ and an external electric field *E*_*ex*_ in the vicinity of a counter electrode.

The self-initiated monomer gas hexafluoropropylene oxide (HFPO) is used together with perfluorobutanesulfonyl flouride (PFBSF) to polymerize PTFE thin films on the cooled substrate. The chemical structures of these two molecules are shown in Fig. 1b. The films are deposited on conductive substrates, which act as bottom electrodes. Due to the mild deposition conditions, also flexible polymer substrates can be coated, as demonstrated in Fig. 1c. The iCVD PTFE thin films are subsequently irradiated with ions in a corona discharge setup, as schematically illustrated in Fig. 2a.

The characteristic parameters of an electret are illustrated in Fig. 2b. The resulting surface charge of the electret (*σ*_*s*_) after the ion irradiation generates an internal electric field (*E*_*in*_) and an external electric field (*E*_*ex*_) in the vicinity of a counter electrode. It is related to the surface potential (*V*_*S*_) of the electret by:

1 $${V}_{S}(t)=\frac{{\sigma }_{S}(t){x}_{1}}{{\varepsilon }_{0}{\varepsilon }_{r}}.$$

**Figure 3:**
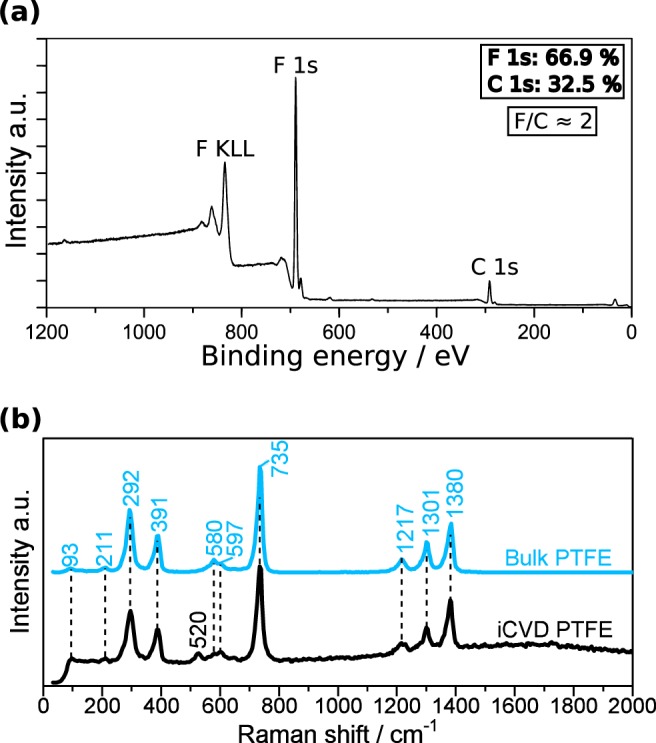
**(a)** XPS spectrum of the iCVD PTFE thin film showing the characteristic CF_2_ ratio for PTFE. **(b)** Identical bands can be observed in the Raman measurement of the iCVD thin film and a commercial bulk PTFE reference sample.

The value *x*_1_ represents the film thickness and ε_0_ and ε_r_ denote the vacuum permittivity and relative permittivity, respectively. The initial value of *σ*_*s*_ is limited by the corona parameters and dielectric strength of the material. Non charged iCVD PTFE films deposited on C-Si are used for the chemical characterization. The similarity of iCVD PTFE films to commercial PTFE is reported by many authors^22,24^. Also the X-ray photoelectron spectroscopy (XPS) and Raman spectroscopy conducted for this study confirm the similarity of iCVD PTFE thin films to commercial bulk PTFE, as shown in Fig. 3.

**Figure 4:**
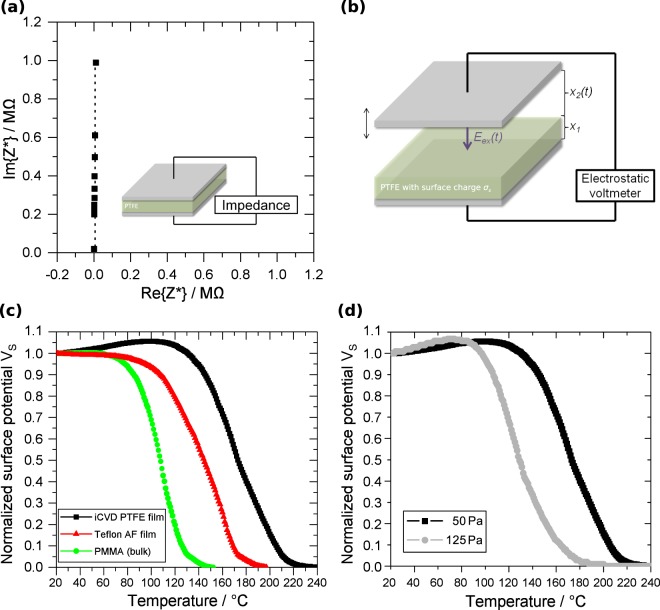
**(a)** Nyquist plot of iCVD PTFE thin film measured in the thin film capacitor arrangement. **(b)** Schematic sketch of the electrostatic voltmeter used for the contact-free measurement of the electret with a vibrating probe. **(c)** TSPD measurement of iCVD PTFE, PMMA and commercial Teflon AF thin film electrets at 6 °C min^−1^ heating rate. The error bars are within the symbol size. **(d)** TSPD curves for iCVD PTFE deposited at two different reactor pressures show different behavior during the measurement (heating rate 6 °C min^−1^ for both samples). The error bars are within the symbol size.

The XPS spectrum in Fig. 3a shows a C 1 s peak around 292 eV, which can be assigned to the carbon in the backbone of the molecule^25^. The peak around 690 eV is identified as the primary F 1 s peak for the fluorine atoms, bound to the carbon backbone, while the peaks in the region between 800 eV and 900 eV can be associated with the Auger peak for fluorine (F KLL)^25^. Considering the sensitivity factor 5.1 for F 1 s, a concentration of 66.9% for F 1 s and 32.5% for C 1 s can be estimated implying the characteristic F/C ≈ 2 ratio for PTFE. Further information on the high resolution C 1s peak can be found in the supplementary information (Fig. S1). Recorded Raman spectra of iCVD PTFE thin films and bulk PTFE show identical bands, as shown in Fig. 3b. A detailed band assignment can be found in the supplementary data. The 520 cm^−1^ band appears only in the iCVD PTFE thin film spectrum. Due to the absence of A2 vibrations in Raman spectroscopy it can not be attributed to the CF_2_ rocking which is typically observed in this range in IR spectra of PTFE^26,27^. But a typical strong band around 520 cm^−1^ is associated with the main one-phonon peak in c-Si^28,29^ and the band at 520 cm^−1^ is thus identified as a substrate peak for the c-Si substrate. Other than that, the two spectra for bulk PTFE and iCVD PTFE show excellent agreement and the obtained PTFE thin films seem to be identical to commercial bulk PTFE. The influence of the corona discharge on the polymer surface morphology has been investigated in detail before, e.g. by Kim *et al*.^30^. The conformity of the iCVD PTFE films grown on c-Si is investigated by ellipsometry and shows a value of 18.0 ± 5.5 nm thanks to the CVD-typical growth characteristics. The dielectric properties of the iCVD deposited films are determined by the impedance, measured in a thin film capacitor arrangement. As shown in Fig. 4a, the vertical line in the Nyquist plot represents capacitive behavior.

The relative permittivity of the iCVD PTFE is determined from the complex impedance Z* by

2 $${{\varepsilon }_{r}}^{\ast }={(i\omega {{\rm{C}}}_{0}{Z}^{\ast })}^{-1}$$

The variable *ω* represents the angular frequency and C_0_ the geometry factor. The relative permittivity is determnined to ε_r_ = 2.1, which is typically reported for PTFE^31^. The dissipation factor is given by:

3 $$\tan (\delta )=\frac{{\varepsilon }_{2}}{{\varepsilon }_{1}}.$$

It is calculated for commercial PTFE and iCVD PTFE from the impedance data. The value *ε*_2_ represents the imaginary part and *ε*_1_ represents the real part of the complex relative permittivity (*ε*_r_* = *ε*_1_ + *iε*_2_). In the setup used in this study, no difference between iCVD PTFE and commercial PTFE was found. This confirms that iCVD PTFE does not only resemble commercial PTFE with regard to chemical properties, but further on with regard to the dielectric properties. Due to the CF_3_ end-groups, provided by the PFBSF initiator, the dissipation factor is much lower as typically observed in, e.g. plasma polymerized PTFE thin films^18^, because polar C=O group formation can be excluded. The corona charged iCVD PTFE electrets are investigated with regard to their charge stability with an electrostatic voltmeter (Fig. 4b). Thermally stimulated potential decay (TSPD) measurements are performed for this purpose. The thermal charge stability of the iCVD PTFE thin film electrets is compared to conventional spin coated Teflon AF thin film electrets^32,33^ and PMMA. The results of the measurements are shown in Fig. 4c. The measurement shows that fluoropolymers, like iCVD PTFE and Teflon AF, are generally superior to non-fluoropolymers, as already mentioned in the beginning. The iCVD PTFE thin films show even higher charge stability compared to conventional Teflon AF fluoropolymer thin films. A factor, which might decrease the performance of the Teflon AF is the preparation method by spin coating. Residual solvent might be embedded in the spin coated Teflon AF film and inhomogeneous film quality might occur, because multiple layers have to be spin coated in order to obtain a sufficient high film thickness for reasonable electret charge storage. Furthermore the amorphous Teflon AF undergoes a glass transition around T_g_ = 160 °C. The surface potential increase of iCVD PTFE, observed up to 120 °C, can be associated with the thermal expansion of the thin film^34^. It is very pronounced in PTFE due to the phase transition in the crystalline regions from hexagonal (15/7 helix) to a pseudohexagonal disordered structure at 30 °C^35^. The thermally stimulated current *dn/dt* released from the iCVD PTFE electrets at heating rate *β* can be calculated from *V*_*S*_ by^36^:

4 $$\frac{dn}{dt}=\frac{{\varepsilon }_{0}{\varepsilon }_{r}}{x}\beta \frac{d{V}_{S}}{dT}.$$

The plot can be found in the supplementary information (Fig. S2). Let *E* be the trap energy level. The function *f*_0_*(E)* represents the initial occupancy of the traps and *N(E)* the energy distribution of the trap levels, the product *f*_0_*(E)N(E)* thus the initial number density^37,38^. With *P(E*, *T)* as a shape-factor, the carrier current can be expressed, according to Simmons *et al*., by^34,39,40^:

5 $$\frac{dn}{dt}={\int }_{{E}_{0}}^{E}\,{f}_{0}(E)N(E){\rm{P}}({\rm{E}},\,{\rm{T}})dE.$$

Shape-factor *P(E*, *T)* in this connection is given by:

6 $$P(E,\,T)=e(E,\,T)\exp (-\frac{1}{\beta }{\int }_{{T}_{0}}^{T}\,e(E,\,T)dT).$$

The function *e(E*, *T)* is the release probability including the attempt-to-escape frequency *ω* and can be described by:

7 $$e(E,\,T)=\omega \,\exp (-\frac{E}{kT}).$$

*P(E*, *T)* can be approximated by a *δ*-function considering the associated area *D* (see^39^ for more details) by:

8 $$P(E,\,T)=D\delta (E-{E}_{max}).$$

This reduces (5) to:

9 $$\frac{dn}{dt}\cong D{f}_{0}(E)N({E}_{max}),$$

with *dP(E*, *T)/dE* = 0, Simmons *et al*. suggested the solution

10 $$E=T(a\,\mathrm{log}(\frac{\omega }{\beta })+b)-c$$

with parameters a = 1.92 × 10^−4^, b = 3.2 × 10^−4^ and c = 1.5 × 10^−2^. From this equation, *ω* can be estimated by measuring the temperature of the current peak at two different heat rates (β_1_ = 3.75 °C min^−1^ and β_2_ = 6 °C min^−1^). The determined value of 3.6 × 10^13^ Hz for iCVD PTFE shows excellent agreement to reported values for commercial PTFE (7 × 10^13^ Hz)^34^. The activation energy of the traps is estimated to *E*_*a*_ = 1.163 eV. The iCVD technique enables furthermore to tune these properties by a change in the deposition parameters. They have a direct influence on the underlying free radical polymerization and hence the resulting structural properties, like molecular weight and crystallinity of the PTFE thin film. Fig. 4d shows two iCVD PTFE thin film electrets with identical film thickness (13 µm) grown at different process pressures. The reduction in mean free path during the reaction (125 Pa) is accompanied with a decrease in thermal charge stability, as seen in Fig. 4d. The iCVD enables thus a direct control of the charge trapping behavior in the polymer film via the deposition parameters. This might be used to tune the charge trapping of the electret layer in e.g. electret memristors^8^ or similar applications. The presented results show that the iCVD technique is well applicable for the production of PTFE thin films for electret applications thanks to their excellent resemblance to commercial PTFE with regard to chemical and dielectric properties. The mild deposition conditions enable furthermore the deposition on temperature sensitive substrates like flexible organic substrates for next generation organic electret devices. The conformal growth and easy scale-up allows furthermore an easy integration into state-of-the-art microelectronic processing lines and can be considered for next generation fluoropolymer thin film electret fabrication.

## Methods

### Fabrication of PTFE thin films

PTFE thin films were grown on aluminium and crystalline silicon (c-Si, Si-Mat) substrates by iCVD. The system was operated in continuous flow mode. Perfluorobutanesulfonyl flouride (PFBSF, 95% Fluorochem, UK) was mixed with hexafluoropropylene oxide (HFPO, 97%, Fluorochem, UK) at a ratio of 1/5. The HFPO flow was controlled by a mass flow controller (Alicat Scientific MC series). PFBSF was kept in a glass jar at room temperature and the PFBSF vapor was delivered through a leak valve (Varian) to the reactor. The overall flow was 1.2 sccm. The filament power of 50 W was applied during the deposition by a power supply (Knürr-Heinzinger), using a Nickel Chromium (Ni80/Cr20, Goodfellow GmbH) filament array. The sample stage was cooled to 20 °C by a circulating thermostat (Huber CC-K6). The process pressure of 50 Pa inside the reactor was controlled by a downstream butterfly valve (VAT Series 615), coupled to a capacitance manometer (MKS Baratron), which was attachted to the reactor. Additional sample sets of identical film thickness were produced using different process pressures (125 Pa and 25 Pa) to study the influence of pressure on the charge storage behavior of the polymer film.

### Film characterization

The film thickness for the iCVD PTFE samples was obtained by profilometer measurements (Bruker Dektak XT). X-ray photoelectron spectroscopy (XPS) were performed using a UHV system equipped with a hemispherical electron analyser and a twin anode–x-ray source (Omicron Full Lab, Omicron Nano- Technology GmbH). An Al Kα X-ray source at a power of 240 W is used in the system. CasaXPS software was used to perform a quantitative analyses and detailed peak deconvolution. Raman spectroscopy is performed with a confocal Raman spectroscopy microscope (WITec Alpha300 RA). The laser excitation line is 532 nm, using a power of 5 mW in the range of 80–2000 cm^−1^ with spectral resolution 5 cm^−1^. The same measurements are performed for commercial bulk PTFE (Goodfellow). Further measurements were performed with a variable-angle spectroscopic ellipsometry (M-2000UI, J.A. Woollam Co., Inc.) in reflection mode at four angles (55°, 60°, 65° and 70°). The optical data has been recorded from 250 nm to 1700 nm. The resulting data was processed with the CompleteEASE software. The system was modeled with one layer for the c-Si substrate and a Cauchy layer for the polymer film.

### Electret fabrication

13 µm iCVD PTFE films were grown on 0.5 mm aluminum. A custom-made point-to-plane DC corona discharge setup is operated at *V*_*tip*_ = −6.5 kV tip potential and used to irradiate the samples with ions in order to fabricate electrets. The discharge is performed in ambient air for 60 s. The tip-to-sample distance is 5 mm. In this way iCVD PTFE thin film electrets, spin coated Teflon AF (Du Pont) electrets and bulk PMMA (Goodfellow) electrets are fabricated.

### Dielectric and electret characterization

The phase angle and absolute value of the impedance of the non-charged iCVD PTFE samples were measured from 100 Hz to 1 MHz in a custom-made parallel plate setup containing a programmable LCR meter (Fluke PM6306). The surface potential of the charged electret samples was determined by an electrostatic voltmeter (Trek 347). The probe was mounted over a grounded, heatable stainless steel plate. Thermally stimulated potential decay (TSPD) measurements were performed by recording temperature and surface potential. Heating rates of 3.75 °C min^−1^ and 6 °C min^−1^ were used. The computation of all presented results were performed in Matlab R2016a.

## Supplementary information

Supplementary Information

## References

[1] Xia Z, Wedel A, Danz R (2003). Charge storage and its dynamics in porous polytetrafluoroethylene (PTFE) film electrets. IEEE Trans. Dielectr. Electr. Insul..

[2] Remke RL, von Seggern H (1983). Modeling of thermally stimulated currents in polytetrafluoroethylene. J. Appl. Phys..

[3] Sessler, G. M. Electrets. 2, 13–75. (Springer Berlin Heidelberg, 1987).

[4] Sessler GM, West JE (1962). Self-Biased Condenser Microphone with High Capacitance. J. Acoust. Soc. Am..

[5] Sessler GM, West JE (1073). Electret transducers: a review. J. Acoust. Soc. Am..

[6] Zhong J (2016). Surface charge self-recovering electret film for wearable energy conversion in a harsh environment. Energy Environ. Sci..

[7] Baeg K-J (2006). Organic Non-Volatile Memory Based on Pentacene Field-Effect Transistors Using Polymeric Gate Electret. Adv. Mater..

[8] Zhong Y-N, Wang T, Gao X, Xu J-L, Wang S-D (2018). Synapse-Like Organic Thin Film Memristors. Adv. Funct. Mater..

[9] Gong S (2018). Monocharged Electret Generator for Wearable Energy Harvesting Applications. Adv. Sustainable Syst..

[10] Hu F, Cai Q, Liao F, Shao M, Lee S-T (2015). Recent Advancements in Nanogenerators for Energy Harvesting. Small.

[11] Wang B (2016). Sandwiched Composite Fluorocarbon Film for Flexible Electret Generator. Adv. Electron. Mater..

[12] Chiu Y, Wu S-H (2013). Flexible electret energy harvesters with parylene electret on PDMS substrates. J. Phys.: Conf. Ser..

[13] Huiming X, Gangjin C, Xumin C, Zhi C (2017). A Flexible Electret Membrane with Persistent Electrostatic Effect and Resistance to Harsh Environment for Energy Harvesting. Sci. Rep..

[14] Kacprzyk R, Ziaja J (1997). Properties of corona charged plasma vapour deposited PTFE film. J. Electrostat..

[15] Amyot N, Klemberg-Sapieha JE, Wertheimer MR, Ségui Y, Moisan M (1992). Electrical and structural studies of plasma-polymerized fluorocarbon films. IEEE Trans. Electr. Insul..

[16] Schwödiauer R (1998). Charge stability of pulsed-laser deposited polytetrafluoroethylene film electrets. Appl. Phys. Lett..

[17] Morrison DT, Robertson T (1973). R.F. sputtering of plastics. Thin Solid Films.

[18] Hetzler U, Kay E (1978). Conduction mechanism in plasma-polymerized tetrafluoroethylene films. J. Appl. Phys..

[19] Wang M (2017). CVD Polymers for Devices and Device fabrication. Adv. Mat..

[20] Chen N (2016). Polymer Thin Films and Surface Modification by Chemical Vapor Deposition: Recent Progress. Annu. Rev. Chem. Biomol. Eng..

[21] Pryce Lewis HG, Caulfield JA, Gleason KK (2001). Perfluorooactane Sulfonyl Fluoride as and Initiator in Hot-Filament Chemical Vapor Deposition of Fluorocarbon Thin Films. Langmuir.

[22] Lau KKS, Murthy SK, Pryce Lewis HG, Caulfield JA, Gleason KK (2003). Fluorocarbon dielectrics via hot filament chemical vapor deposition. J. Fluor. Chem..

[23] Pryce Lewis HG, Bansal NP, White AJ, Handy ES (2009). HWCVD of polymers: Commercialization and scale-up. Thin Solid Films.

[24] Coclite AM (2013). 25th Anniversary Article: CVD Polymers: A New Paradigm for Surface Modification and Device Fabrication. Adv. Mater..

[25] Ackeret M (1992). Polytetrafluoroethylene by XPS. Surf. Sci. Spectra.

[26] Moynihan RE (1959). The Molecular Structure of Perfluorocarbon Polymers. Infrared Studies on Polytetrafluoroethylene. J. Am. Chem. Soc..

[27] Peacock CJ, Hendra PJ, Willis HA, Cudby MEA (1970). Raman spectrum and vibrational assignment for poly(tetrafluoroethylene). J. Chem. Soc. A..

[28] Uchinokura K, Sekine T, Matsuura E (1972). Raman scattering by silicon. Solid State Commun..

[29] Russell JP (1965). Raman Scattering in Silicon. Appl. Phys. Lett..

[30] Kim CY, Goring DAI (1971). Surface morphology of polyethylene after treatment in a corona discharge. J. Appl. Polym. Sci..

[31] Koizumi N, Yano S, Tsuji F (1968). Dielectric properties of polytetrafluoroethylene and tetrafluoroethylene-hexafluoropropylene copolymer. J. Polym. Sci. C.

[32] Günther, P., Ding, H. & Gerhard-Multhaupt, R. Electret properties of spin-coated Teflon AF films *IEEE Conf. Electr. Insul. Dielectr. Phenomena*, 197–202 (1993).

[33] Hirschberg RE (2018). Electret films with extremely high charge stability prepared by thermal evaporation of Teflon AF. Org. Electron..

[34] Rychkov, D., Gerhard, R., Kuznetsov, A. & Rychkov, A. Modeling the isothermal charge decay of modified polytetrafluoroethylene electrets from their thermally stimulated discharge *IEEE Conf. Electr. Insul. Dielectr. Phenomena*, 105–108 (2016).

[35] Brown EN, Dattelbaum DM (2005). The role of crystalline phase on fracture and microstructure evolution of polytetrafluorothylene (PTFE). Polymer.

[36] Thyssen A, Almdal K, Thomsen EV (2017). Electret stability related to the crystallinity in polypropylene. IEEE Trans. Dielectr. Electr. Insul..

[37] Simmons JG, Taylor GW (1972). High-Field isothermal currents and thermally stimulated currents in insulators having discrete trapping levels. Phys. Rev. B.

[38] Simmons JG, Tam MC (1973). Theory of isothermal currents and the direct determination of trap parameters in semiconductors and insulators containing arbitrary trap disctributions. Phys. Rev. B.

[39] Simmons JG, Taylor GW, Tam MC (1973). Thermally Stimulated Currents in Semiconductors and Insulators Having Arbitrary Trap Distributions. Phys. Rev. B..

[40] Rychkov AA, Cross GH, Conchart MG (1992). Charge relaxation in structures containing non-polar polymer-metal interfaces. J. Phys. D: Appl. Phys..

