# Supplementary material for: Tunable polytetrafluoroethylene electret films with extraordinary charge stability synthesized by initiated chemical vapor deposition for organic electronics applications
Source: Sci Rep. 2019 Feb 19;9:2237. doi: 10.1038/s41598-018-38390-w (PMC6381081; doi:10.1038/s41598-018-38390-w)
Supplement: Supplementary file 1 — Supplementary Information [file 41598_2018_38390_MOESM1_ESM.pdf]

Supplementary Information

Tunable polytetrafluoroethylene electret films with extraordinary charge stability synthesized by initiated chemical vapor deposition for organic electronics applications

Stefan Schröder[1]*, Thomas Strunskus*[1]*, Stefan Rehders*[1]*, Karen K. Gleason*[2]*, and Franz Faupel*[1]*

[1]Institute for Materials Science, Christian-Albrechts-Universität zu Kiel, 24143 Kiel, Germany

[2]Department of Chemical Engineering, Massachusetts Institute of Technology, Cambridge, MA 02139, USA

Table S1: Band assignment for the Raman spectra of bulk PTFE and iCVD PTFE shown in figure 2b.

Raman shift / cm^{-1}	Symmetry	Assignment	Reference
93	E_1	δCC (skeletal torsion)	[27]
211	E_1	τCF_2 (CF_2 twist)	[27]
292	A_1	τCF_2 (CF_2 twist)	[27]
391	A_1	δCF_2 (CF_2 deformation)	[27]
520		c-Si Substrate peak	[28], [29]
580	E_1	pCF_2 (CF_2 rocking)	[27]
735	A_1	νCC (C-C symmetric stretch)	[27]
1217	E_1	$\nu_{as}CF_2$ (CF_2 asymmetric stretch)	[27]
1301	E_2	$\nu_{s}CF_2$ (CF_2 symmetric stretch)	[27]
1380	A_1	$\nu_{s}CF_2$ (CF_2 symmetric stretch)	[27]

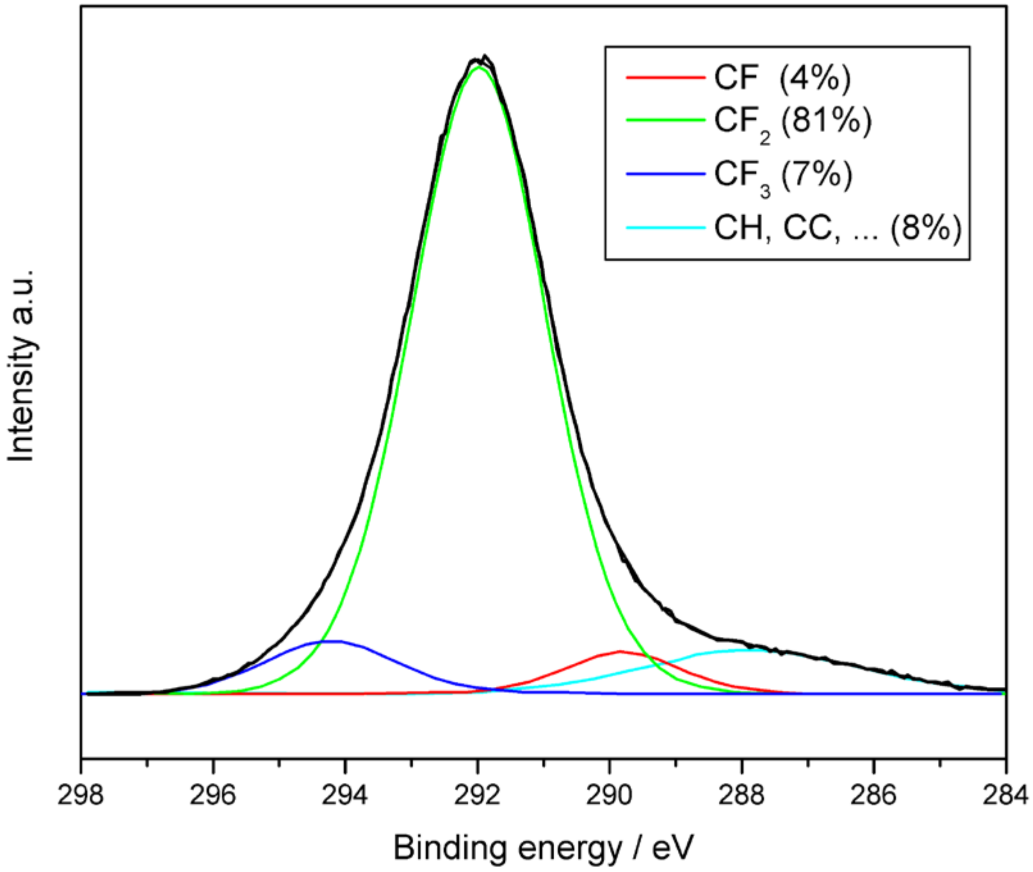

Figure S1 High resolution XPS C1s peak of iCVD PTFE thin films. The CF_2 character of the samples can be seen. There is also a small contribution from CF_3, probably due to the PFBSF initiator surface condensation, as well as PTFE polymer chain end-groups. CH, CC is most likely present due to slight surface contamination of the sample.

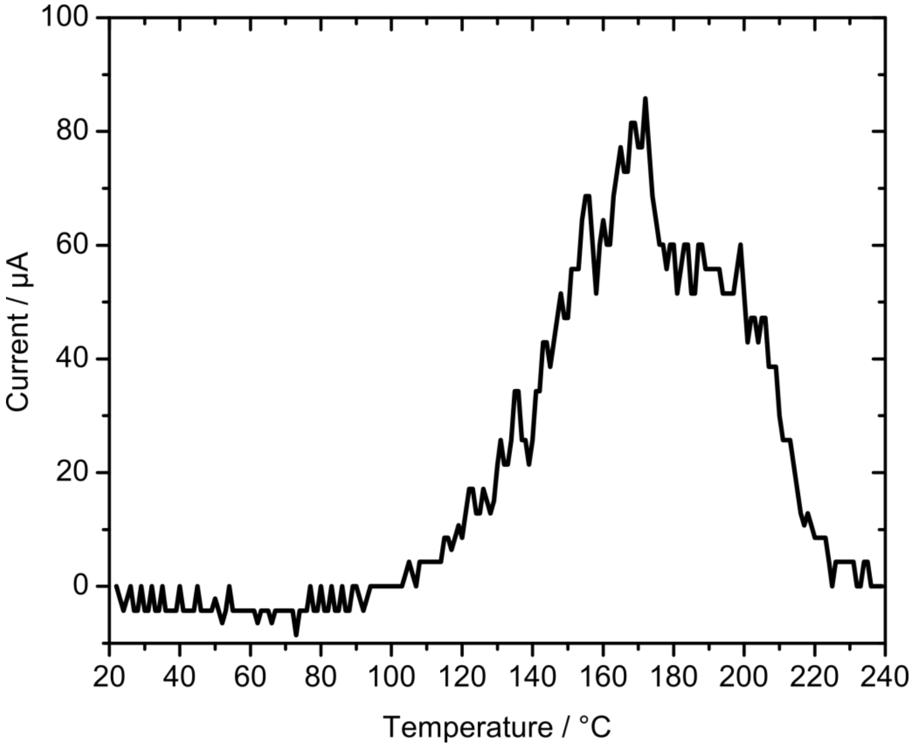

Figure S2 Theoretically calculated TSD curve for iCVD PTFE thin films calculated by equation (4).
